# Identification of Alternative Polyadenylation in *Cyanidioschyzon merolae* Through Long-Read Sequencing of mRNA

**DOI:** 10.3389/fgene.2021.818697

**Published:** 2022-01-28

**Authors:** Leonard Schärfen, Dagmar Zigackova, Kirsten A. Reimer, Martha R. Stark, Viktor A. Slat, Nancy J. Francoeur, Melissa L. Wells, Lecong Zhou, Perry J. Blackshear, Karla M. Neugebauer, Stephen D. Rader

**Affiliations:** ^1^ Department of Molecular Biophysics and Biochemistry, Yale University, New Haven, CT, United States; ^2^ Department of Chemistry, University of Northern British Columbia, Prince George, BC, Canada; ^3^ Department of Genetics and Genomic Sciences, Icahn School of Medicine at Mount Sinai, New York, NY, United States; ^4^ The Signal Transduction Laboratory, National Institute of Environmental Health Sciences, National Institutes of Health, Research Triangle Park, Durham, NC, United States; ^5^ Integrative Bioinformatics Support Group, National Institute of Environmental Health Sciences, National Institutes of Health, Research Triangle Park, Durham, NC, United States

**Keywords:** *Cyanidioschyzon merolae*, alternative polyadenylation, splicing, intron retention, cleavage and polyadenylation, polyadenylation (polyA) signal, polyadenylation site (PAS), nitrogen stress

## Abstract

Alternative polyadenylation (APA) is widespread among metazoans and has been shown to have important impacts on mRNA stability and protein expression. Beyond a handful of well-studied organisms, however, its existence and consequences have not been well investigated. We therefore turned to the deep-branching red alga, *Cyanidioschyzon merolae*, to study the biology of polyadenylation in an organism highly diverged from humans and yeast. *C. merolae* is an acidothermophilic alga that lives in volcanic hot springs. It has a highly reduced genome (16.5 Mbp) and has lost all but 27 of its introns and much of its splicing machinery, suggesting that it has been under substantial pressure to simplify its RNA processing pathways. We used long-read sequencing to assess the key features of *C. merolae* mRNAs, including splicing status and polyadenylation cleavage site (PAS) usage. Splicing appears to be less efficient in *C. merolae* compared with yeast, flies, and mammalian cells. A high proportion of transcripts (63%) have at least two distinct PAS’s, and 34% appear to utilize three or more sites. The apparent polyadenylation signal UAAA is used in more than 90% of cases, in cells grown in both rich media or limiting nitrogen. Our documentation of APA for the first time in this non-model organism highlights its conservation and likely biological importance of this regulatory step in gene expression.

## Introduction

Splicing and polyadenylation are key events required for the maturation of precursor protein coding RNAs to functional mRNAs. Both processes have the potential to generate diverse mRNA isoforms from the same RNA precursor, altering their protein-coding potential and/or their stability (Mayr, 2019; Zhang et al., 2021). Together with transcriptional regulation, these major RNA processing mechanisms thereby underlie differences between the transcriptomes and proteomes expressed during cell differentiation and development in all eukaryotic species. Splicing greatly influences the gene output and is tightly regulated by other pre-mRNA processing steps, such as the transcription elongation and 5′ end capping (Carrocci & Neugebauer, 2019). Coupling between co-transcriptional splicing and polyadenylation cleavage has recently been demonstrated in budding and fission yeasts as well as mouse cells (Herzel 2018; Alpert et al.,2020; Reimer et al., 2021). In fission yeast, transcriptional read-through is antagonized by the nuclear exosome, which degrades transcripts that are not spliced co-transcriptionally (Zhou et al., 2015; Herzel et al., 2018). Thus, splicing can alter the mRNA levels and is coupled to polyadenylation, including cleavage at the polyA site (PAS) and the addition of a 3′ polyA tail. In most organisms, protein coding transcripts are only exported to the cytoplasm when splicing is completed and initial polyA tails are installed.

The critical roles of mRNA polyadenylation include protection from degradation, export of the mature mRNA to the cytoplasm, and facilitation of translation (Jalkanen et al., 2014). Most eukaryotic genes studied so far contain more than one PAS and therefore produce isoforms of mRNAs in a process called alternative polyadenylation (APA). Systematic mapping of PAS’s estimates that up to 80% of mammalian, 75% of *Drosophila*, and more than 80% of yeast genes harbor alternative PAS and display APA (Derti et al., 2012; Hoque et al., 2013; Liu, Freitas, et al., 2017; Liu, Hoque, et al., 2017). APA sites, sometimes also called cryptic sites, can be divided into two categories based on their location relative to the stop codon (Rehfeld et al., 2013). The majority of APA sites are located in the 3′ untranslated region (3′UTR), and the selection of a particular PAS proximal or distal to the stop codon determines the length of the 3′UTR. The length of the 3′UTR and the presence or absence of regulatory sequence affects the stability of the transcripts and protein translation (Ren et al., 2020). However, APA sites can also be found in the introns or exons within the coding region and, in these cases, the PAS selection generates shortened protein isoforms (Ren et al., 2020).

Despite the pivotal role of splicing and APA in gene regulation, these processes have been studied in depth in only in a handful of species. Investigating other organisms could bring important insights, especially from species with fewer components involved in these mechanisms. Species adapted to live in vastly different conditions, such as extreme temperatures, are inherently interesting as their study contributes to our understanding of how basic mechanisms are tuned to the environment. Therefore, we explored the transcriptome of *Cyanidioschyzon merolae (C. merolae),* a unicellular red microalga. Extremophilic *C. merolae* grows in a sulfate-rich, acidic (pH 0.2–4) volcanic springs with temperatures as high as 57°C. The organellar and nuclear genomes of *C. merolae* were the first eukaryotic algal genomes to be completely sequenced (Ohta et al., 1998; Ohta et al., 2003; Matsuzaki et al., 2004; Nozaki et al., 2007). With a compact genome of 16.5 Mbp and 5,331 protein-coding genes (4,775 nuclear) and low genetic redundancy, it is considered one of the simplest eukaryotic organisms (Matsuzaki et al., 2004).

The published *C. merolae* genome reported only 27 introns in 26 genes (Matsuzaki et al., 2004). Interestingly, *C. merolae* contains a simplified spliceosome compared to other organisms. The spliceosomal small nuclear ribonucleoproteins (snRNPs) are comprised of five snRNAs (U1, U2, U4, U5 and U6) and about 100 associated spliceosomal proteins in *Saccharomyces cerevisiae* or over 200 associated proteins in humans (Fabrizio et al., 2009; Agafonov et al., 2011). In contrast, only four snRNAs and 45 core splicing proteins were identified in *C. merolae* (Stark et al., 2015; Reimer et al., 2017). The missing spliceosome component is the U1 snRNA and its associated proteins. U1 snRNA is involved in binding to the 5′ splice site prior to splicing and in telescripting, in which U1 snRNA prevents premature mRNA cleavage at cryptic PAS’s (Berg et al., 2012; Oh et al., 2017; Singh & Singh, 2019). This implies the presence of alternative mechanisms mediating 5′ splice site recognition and protection from premature cleavage in *C. merolae*. It also raises the question of whether *C. merolae* displays altered patterns of PAS recognition and cleavage in the absence of U1. The unique simplicity of the *C. merolae* genome and spliceosome provide an advantage over highly complex systems for studies of mRNA processing. Moreover, the crystal structure of the first *C. merolae* core spliceosome factor, CmSnu13, revealed a near identity to its yeast and human orthologs, further emphasizing the suitability of *C. merolae* in studies of the function of the core spliceosome (Black et al., 2016).

Here, we have employed long-read sequencing (LRS) to enable characterization of individual *C. merolae* transcripts, from their 5′ to 3′ ends, allowing us to investigate splicing efficiency and PAS selection for the first time in this organism. *C. merolae* cells were cultivated either in rich media (RM) or in low nitrogen (LN) media to induce nitrogen starvation stress to study the regulation of splicing and APA under stress. Our results indicate a wide range of splicing efficiencies of individual intron-containing genes and overall increased splicing in the LN conditions. We discovered that a majority of *C. merolae* genes undergo APA under the control of highly conserved regulatory sequences.

## Methods

### *C. merolae* Culturing

The 10D strain of *C. merolae* (NIES-3377) was cultured in modified Allen’s autotrophic media with double the amount of trace elements (MA2 = rich media/RM) (Ohnuma et al., 2008) or in MA2 media in which the 40 mM (NH_4_)_2_SO_4_ was replaced with 40 mM Na_2_SO_4_ (low nitrogen media/LN). Cultures were grown in graduated glass cylinders at 42°C in a CO_2_ incubator, with 2% CO_2_ bubbled directly into the cylinders. Cells were grown under continuous light at 90 µmol photons m^−2^ s^−1^. Cells were grown in RM until they reached an OD_750_ of 0.8–1.0. Cultures were centrifuged at 2000xg for 10 min and then washed in either RM or LN before being resuspended in the corresponding media and grown for 1 h (from the time of the wash) before being harvested.

### Total RNA Isolation

*C. merolae* cultures (10 ml) were harvested after 1 h of growth in the selected media. Cell pellets were resuspended in 500 µL cold phenol RNA lysis buffer (0.5 M NaCl, 0.2 M TrisHCl (pH 7.5), 0.01 M EDTA, 1% SDS) and were sonicated briefly to shear the genomic DNA. Total RNA was extracted two times with acid phenol/chloroform (pH 4.5, Ambion), followed by chloroform extraction. RNA was EtOH precipitated and resuspended in dH2O and then treated with Turbo DNase (Ambion) following the manufacturer’s instructions.

### Standard IsoSeq Library Preparation and Sequencing

IsoSeq SMRTbell libraries were prepared as recommended by the manufacturer (Pacific Biosciences). Briefly, 500 nanograms of total RNA from each sample was used as input for cDNA synthesis using the NEBNext Single Cell/Low Input cDNA Synthesis & Amplification Module (NEB, #E6421L), which employs a modified oligodT primer and template switching technology to reverse-transcribe full-length polyadenylated transcripts. Following double-stranded cDNA amplification and purification, the full-length cDNA was used as input into SMRTbell library preparation, using SMRTbell Express Template Preparation Kit v2.0. Briefly, a minimum of 100 ng of cDNA from each sample was treated with a DNA Damage Repair enzyme mix to repair nicked DNA, followed by an End Repair and A-tailing reaction to repair blunt ends and polyadenylate each template. Next, barcoded overhang SMRTbell adapters were ligated onto each template and purified using 0.6X AMPure PB beads to remove small fragments and excess reagents (Pacific Biosciences). The completed SMRTbell libraries were further treated with the SMRTbell Enzyme Clean Up Kit to remove unligated templates and equimolar pooled together. The final libraries were then annealed to sequencing primer v4 and bound to sequencing polymerase 3.0 before being sequenced on one SMRTcell 8M on the Sequel II system with a 30 h movie.

### Custom IsoSeq Library Preparation and Sequencing to Capture Non-polyadenylated Transcripts

To profile non-polyadenylated, full-length RNA molecules, a custom adaptation of the standard IsoSeq protocol was performed. First, polyA polymerase (*E. coli* PolyA Polymerase, NEB, #M0276S) was used to catalyze polyA tailing of the 3′ ends of all RNA templates using 3 μg of total RNA as input and by following the manufacturer’s instructions. Next, to avoid sequencing predominant rRNA molecules, the NEBNext Custom RNA Depletion Tool (NEB) was used to design hybridization probes tiled against known *C. merolae* rRNA sequences. The custom hybridization probes were then synthesized as a ssDNA oligo pool supplied at a concentration of 10 pmol per oligo (Integrated DNA Technologies) and were used in combination with the NEBNext RNA Depletion Core Reagent Set (NEB, #E7865S) to deplete rRNA molecules. Briefly, 1 μg of polyA-tailed RNA per sample was supplied as input to a hybridization reaction with the ssDNA depletion oligos and the targeted molecules were subsequently degraded by RNaseH digestion. Surplus DNA probes were then digested with DNase I treatment and the samples were subsequently washed using 1.8X Agencourt RNACleanXP beads (Beckman Coulter) to remove digested nucleotides and excess reagents. The purified polyA-treated, rRNA-depleted RNA samples were then subjected to SMRTbell library preparation and sequencing as described above.

### IsoSeq Data Primary Processing

After data collection, the raw sequencing subreads were imported to the SMRTLink analysis suite, version 10.1 for processing. The latest version of PacBio’s IsoSeq3 (v.3.4.0) pipeline was used for analysis. First, intramolecular error correcting was performed using the circular consensus sequencing (CCS) algorithm (v.6.0.0) to produce highly accurate (>Q20) CCS reads, each requiring a minimum of three polymerase passes. The CCS reads were then passed to the *lima* (v.2.0.0) tool first to demultiplex samples by barcoded adapters, and second, using the specialized “-isoseq” mode, to remove IsoSeq template-switching oligo sequences, which also orients the isoforms into the correct 5′ to 3′ direction. Reads were aligned to the *C. merolae* genome using minimap2 (Li, 2018) with the long-read/Pacbio-CCS preset and a maximum intron length of 1 kb.

### Calculating Fraction Spliced of PacBio Long Reads

First, PacBio long reads that spanned an intron-containing gene were extracted from mapped data: Mapped reads in BAM format were converted to BED format with bedtools, then intron-spanning reads were identified by overlapping an annotation of *C. merolae* intron coordinates using bedtools intersect with parameters: *wo*, *F* = 0.3, *s* (Quinlan & Hall, 2010; Dale et al., 2011). Spliced reads in BED format were then classified as intron-spanning reads which had a “blockSize” greater than one, indicating the read mapped to two separate regions in the genome. For each intron, the total number of reads spanning the intron and the number of reads which were classified as spliced was tabulated. Fraction spliced was calculated as the number of spliced reads divided by the total number of reads for each intron. Coverage for each intron was defined as the total number of intron-spanning reads.

### Analysis of Alternative Polyadenylation

Individual reads were assigned to a gene by intersecting a list of gene coordinates generated from the annotation in BED format using bedtools intersect with settings *wo*, *s*, *f* = 0.25. Next, the 3′-end coordinate for each aligned read was extracted by parsing BAM files, and assigned a gene based on the previously generated BED file. Read 3′-ends were mapped for each individual transcript by drawing histograms of the 3′-end positions. The find_peaks algorithm from scipy.signal was used with empirically determined height threshold of 4 and minimum peak distance threshold of 18 nt. Peaks were sorted according to their height and the number of peaks was recorded for every transcript. For meta-analysis, signals for individual transcripts were normalized by dividing by the total number of 3′-ends and then summed after aligning at the highest peak. Sequences of the 500 most expressed genes 40 nt upstream of the first highest, second highest and third highest peak were extracted and exported as fasta files for motif analysis with XTREME using the RNA library and default parameters except for the minimum motif length, which was decreased to 4. The length between the polyadenylation signal and the cleavage site was determined by searching for “UAAA” within 40 nt upstream of the highest peak and calculating the distance between the peak coordinate and the search result coordinate.

### Correlating Intron Features With Fraction Spliced

For each previously identified intron in the *C. merolae* genome, intron length, GC content, and gene 3′UTR length were calculated directly from the gene annotation. Branchpoint sequences for each intron were estimated by eye based on sequence homology to known branchpoint sequences, and the distance from the branchpoint “A” to the 3′SS was calculated. For splice site sequence analysis, the MaxEnt webserver was used to calculate a splice site score for the 5′SS (using the sequence 3 nt upstream to 5 nt downstream) and for the 3′SS (using the sequence 20 nt upstream to 3 nt downstream) (Yeo and Burge, 2004). For secondary structure prediction, intron sequences were input into the RNAfold webserver and the output minimum free energy (kcal/mol) was tabulated for each intron (http://rna.tbi.univie.ac.at//cgi-bin/RNAWebSuite/RNAfold.cgi). All features for each intron were tabulated and plotted against the fraction spliced calculated as above.

### Illumina RNA Sequencing and Data Analysis

Total RNA was quantitated with a Qubit fluorometer, and 1 µg of each sample was transcribed to generate cDNA libraries using the TruSeq Stranded mRNA Sample Prep Kit (Illumina Inc, San Diego, CA) following the manufacturers protocol with the following modifications: 10 cycles were used to enrich DNA fragments to minimize the risk of over-amplification and dual-indexed barcode adapters were applied to each library. Libraries were pooled in an equimolar ratio for sequencing on the Illumina HiSeq 4000 System (Illumina Inc., San Diego, CA). The raw Illumina sequencing reads in FASTQ format were evaluated for quality using FastQC v0.11.9 (www.bioinformatics.babraham.ac.uk/projects/fastqc). The Illumina reads for three rich media replicates were mapped to the *C. merolae* 10D reference genome (Matsuzaki et al., 2004; Nozaki et al., 2007), which was obtained from release 46 of Ensembl Plants (Dobin et al., 2012), using STAR v2.7.1a (Dobin et al., 2012). IRFinder v1.2.5 (Middleton et al., 2017) was used to determine the average intron retention ratio for each intron, which was then subtracted from 1 to calculate the fraction of spliced reads.

## Results

### Long-Read Sequencing Reveals Splicing and Alternative Polyadenylation Status of *C. merolae* mRNAs

**FIGURE 1:**
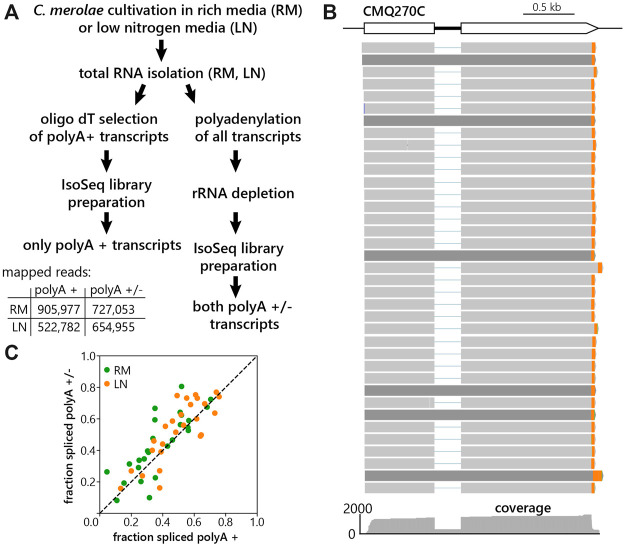
Long-read sequencing of *C. merolae* polyA+ and polyA- RNA. **(A)** Workflow of the experiment with indicated numbers of mapped reads resulting from individual library preparations. **(B)** Long reads aligned to the reference genome, using the intron-containing gene CMQ270 as an example. 3′-ends are marked in orange. **(C)** Correlation of fraction of spliced reads obtained from the polyA+ and polyA+/− libraries prepared from the RM (rich media, green) and LN (low nitrogen, orange) samples of *C. merolae.* Fraction spliced was calculated as the number of intron-spanning reads which were spliced divided by the total number of intron-spanning reads for each gene shown. Pearson’s correlation = 0.80 (RM), 0.82 (LN).

To prepare libraries for LRS, *C. merolae* was cultivated either in RM or LN condition for 60 min. Total RNA was then isolated from both samples, and library preparation proceeded in two different ways (Figure 1.A). First, we used part of the total RNA for reverse transcription using an anchored oligo dT reverse primer, enriching for transcripts with polyA tails on mature mRNAs. Since splicing and polyadenylation are primarily co-transcriptional events, we aimed to also detect precursor RNAs that do not possess polyA tails. Therefore, in the second type of LRS library total RNA was polyadenylated prior to reverse transcription by anchored oligo dT. Thus, we captured both polyA+ and polyA– transcripts. Importantly, ribosomal RNA was removed before reverse transcription. As commercially available kits for the ribosomal depletion did not efficiently remove *C. merolae* rRNA, we designed our own set of probes against *C. merolae* rRNA (Supplementary Table S1). The sequencing on a Pacific Biosciences (PacBio) SMRT cell 8M with the Sequel II system resulted in 905,977 and 522,782 mapped reads for the polyA + libraries and 727,053 and 654,955 reads for the polyA+/− libraries for RM and LN samples respectively (Figure 1A). The reads were mapped to the updated *C. merolae* genome annotation 10D (unpublished). Figure 1B shows that our LRS experiment captures different mRNA isoforms. Comparison of the fraction of spliced reads resulted in a high correlation between the two library preparation strategies (Figure 1C), which indicates that their merge will likely not distort our data analysis. To improve the gene coverage for the downstream data analysis, we therefore combined both polyA+ and polyA ± libraries.

### Alternative Polyadenylation Is Frequent in *C. merolae*

**FIGURE 2:**
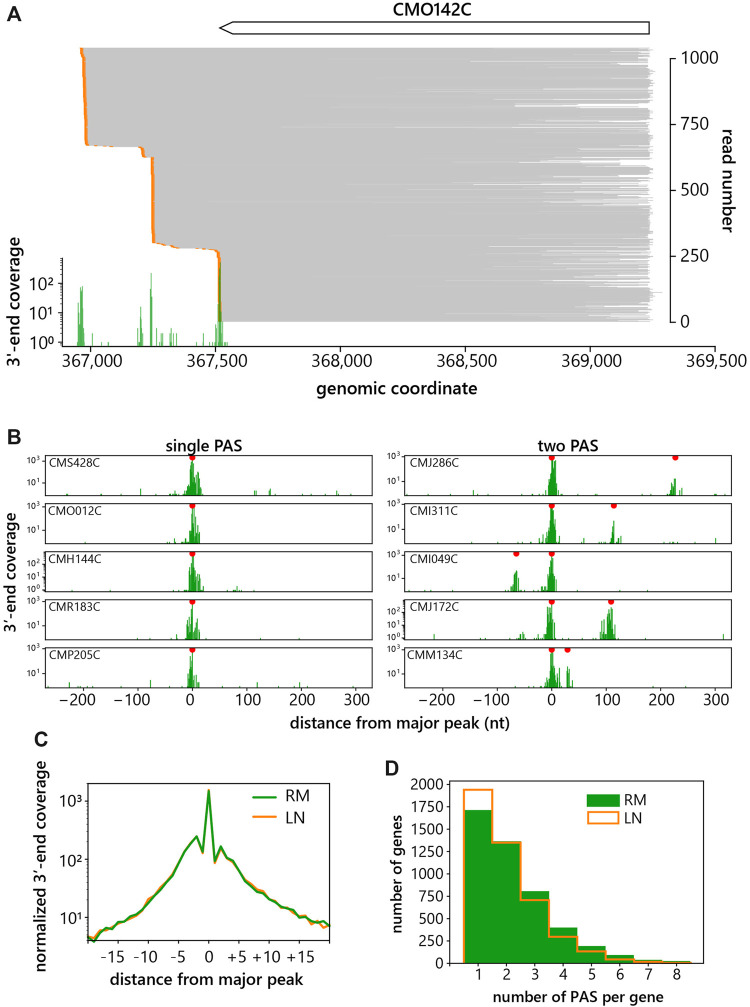
Alternative polyadenylation in *C. merolae.* **(A)** Example of a gene CMO142C with at least four PAS’s showing aligned reads in gray, 3′-ends in orange and a histogram of 3′-end coverage in green. **(B)** Examples of 3′-end coverage distributions for genes with one or two PAS’s. Red dots indicate called peaks that passed the thresholds. **(C)** Metagene analysis of primary PAS peaks. The major peak is at the position of nucleotide 0, RM = rich media, LN = low nitrogen. **(D)** Histogram of the number of PAS’s per gene. RM = rich media, LN = low nitrogen.

To assess the frequency of APA in *C. merolae* we performed a transcriptome-wide analysis of all protein-coding genes. PAS’s were identified by peak calling of read 3′ end positions after trimming of the polyA tails. A peak was considered a PAS when at least four reads ended at the same nucleotide. Figures 2A,B show example of reads mapped to a gene with at least four PAS’s and of 3′end peaks of genes with one or two PAS’s. The metagene analysis of the most prominent peak for each transcript showed cleavage approximately 20 nucleotides to both sides of the primary peak (Figure 2C), indicating that the 3′ end cleavage downstream of a particular polyA signal can occur within a sequence spanning 30–40 nucleotides. Based on this observation the peaks were considered as two different PAS’s if they were at least 18 nucleotides apart. Afterwards, the number of detected peaks with these parameters were counted for each gene. Surprisingly, we detected 63% of genes with more than one PAS and 34% with more than two PAS’s in RM conditions (Figure 2D). Our results indicate that APA is a frequent event in *C. merolae.*

### *C. merolae* Uses UAAA Tetranucleotide as a Polyadenylation Signal

**FIGURE 3:**
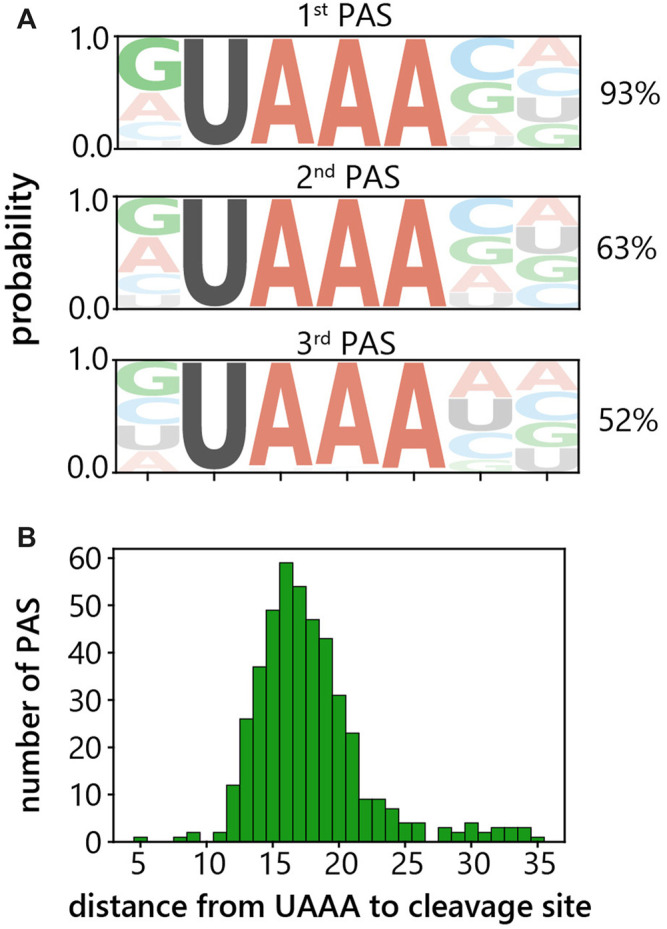
Identification of polyadenylation signal in *C. merolae.* **(A)** Motif analysis of 500 sequences each upstream of primary, secondary and tertiary 3′-end peak. Percentages indicate how many sequences out of the input contained the motif. **(B)** Histogram of the distance between upstream UAAA tetranucleotide and primary cleavage site.

Cleavage of pre-mRNA at the PAS is stimulated by the polyadenylation signal (polyA signal) typically located 10–30 nt upstream of the PAS (Proudfoot, 1991; Colgan & Manley, 1997). Therefore, as our next step, we aimed to identify the polyA signals used in *C. merolae.* To find the motif for the preferentially used PAS’s with the most prominent peaks, upstream sequences were extracted and the motif discovery tool XSTREME was used (Grant and Bailey, 2021). The polyA signal motif for the primary, i.e. most frequently occurring, PAS was found to be UAAA in 93% of genes (Figure 3A). Similarly, the UAAA tetranucleotide was recognized as a polyA signal motif also for the secondary and tertiary PAS’s, in 63 and 52% of genes, respectively (Figure 3A). We did not identify any other significant consensus motifs among the sequences that did not contain UAAA. The distance between the polyA signal and PAS is in the range of 3–35 nucleotides with an average of 17 nucleotides (Figure 3B). These results indicate that the UAAA tetranucleotide serves as a polyA signal in *C. merolae.*

### Intron-Containing Genes in *C. merolae* Display a Wide Variety of Splicing Efficiencies.

**FIGURE 4:**
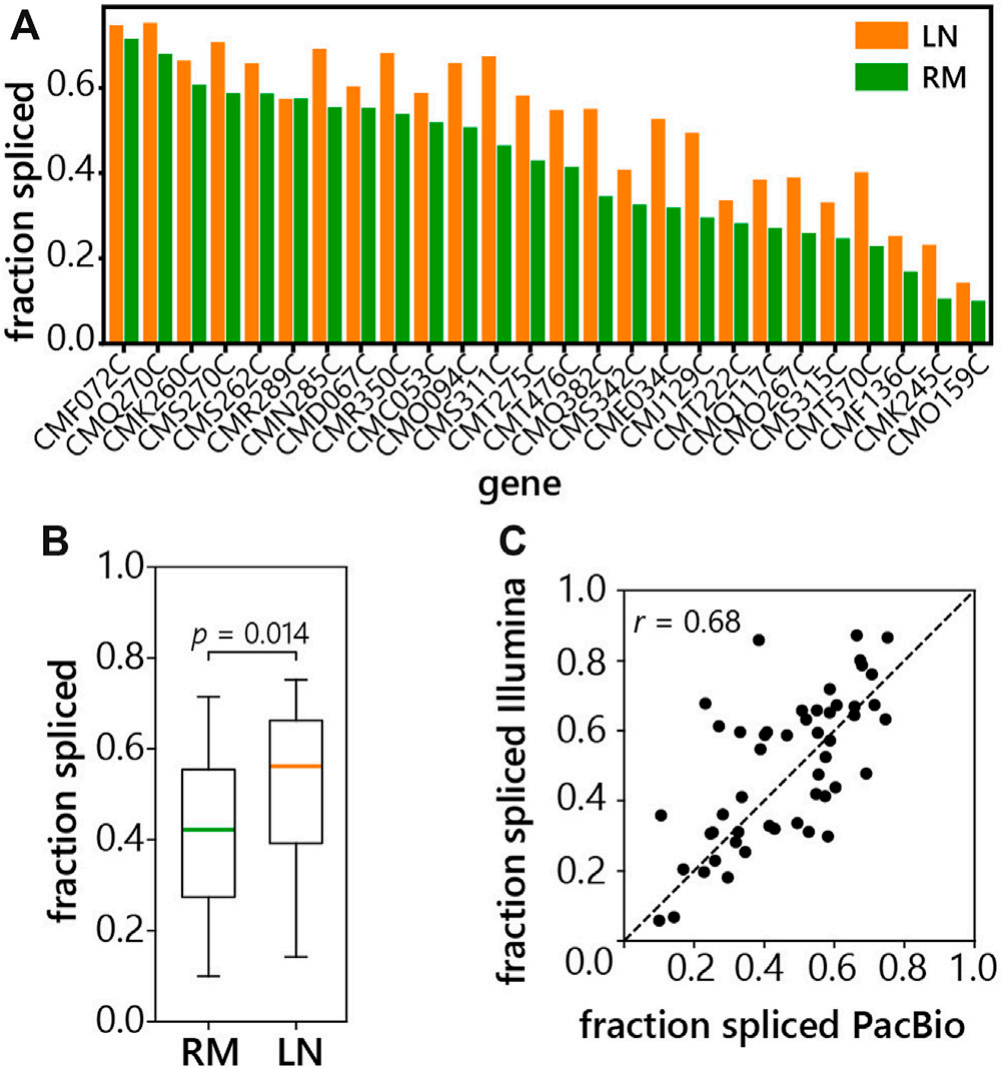
Splicing efficiency of *C. merolae* intron-containing genes. **(A)** LRS was used to measure the fraction of reads that were spliced at each intron-containing gene in *C. merolae*, fraction spliced was calculated as in Figure 1B (see above), LN (low-nitrogen, orange), RM (rich media, green). **(B)** Box plot of a fraction of spliced reads grown in RM or LN. Significance of the difference between fraction of spliced reads was tested by two-sided Mann-Whitney U test. **(C)** Correlation of fraction of spliced reads obtained from the short-read (Illumina) and long-read (PacBio) libraries prepared from samples of *C. merolae.* Fraction spliced was calculated as the number of intron-spanning reads which were spliced divided by the total number of intron-spanning reads for each gene shown. Pearson’s correlation = 0.68.

We calculated the fraction of spliced reads for each of the 26 genes annotated as intron-containing (Matsuzaki et al., 2004). The number of spliced reads was divided by the total number of intron-spanning reads for each gene. Surprisingly, *C. merolae* genes display a wide spectrum of splicing efficiencies from about 10 to 70% spliced reads (Figure 4A). This indicates the presence of a large proportion of transcripts with retained introns that contain polyA tails. In addition, splicing is almost uniformly increased in the LN condition, suggesting stress-induced splicing up-regulation (Figures 4A,B). Though subtle in most cases, the increase in the overall spliced fraction in the LN condition is statistically significant with a *p*-value of 0.014 (Figure 4B). Importantly, the comparison between our LRS data and Illumina short-read sequencing data obtained from the same *C.merolae* samples showed good correlation (Figure 4C). Thus, the splicing efficiency was confirmed by two independent approaches.

**FIGURE 5:**
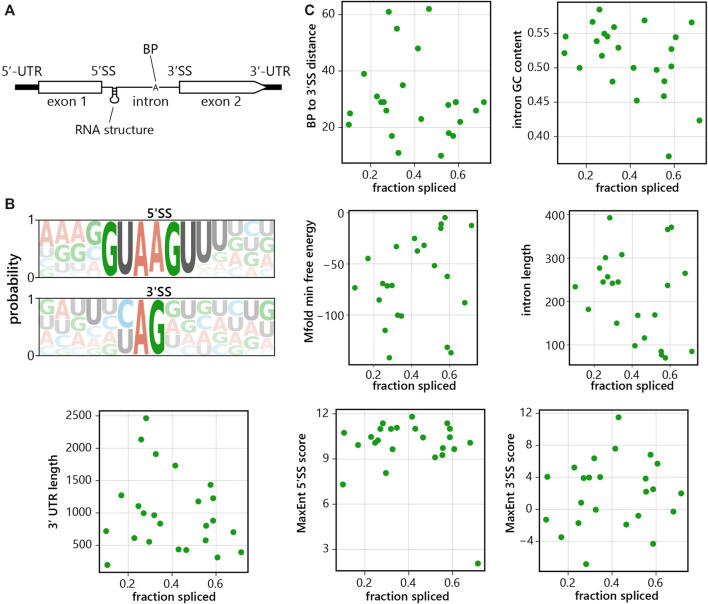
*C. merolae* intron features do not reliably predict splicing efficiency. **(A)** Scheme of intron-containing gene, UTR = untranslated region, SS = splice site, BP = branch point. **(B)** 5′splice site (5′SS) and 3′splice site (3′SS) consensus sequences of *C. merolae* introns. **(C)** Intrinsic features of each intron in *C. merolae* were analyzed to determine their correlation with splicing efficiency (as calculated above, Figure 1B). Each point represents a single intron. Features analyzed included: branchpoint to 3′SS distance (Pearson’s correlation (*r*) = −0.148, *p* = 0.489), 3′SS score (*r* = 0.118, *p* = 0.582), 5′SS score (*r* = -0.237, *p* = 0.264), GC content (*r* = −0.413, *p* = 0.045), predicted secondary structure (*r* = 0.408, *p* = 0.048), intron length (*r* = -0.421, *p* = 0.040), and gene 3′UTR length (*r* = −0.191, *p* = 0.371).

Next, we aimed to analyze individual intron features (Figure 5A) to see if they predict splicing efficiency (Figures 5B,C). We calculated 3′ splice site (using nucleotides -20:+3) and 5′ splice site (using nucleotides -3:+5) scores, reflecting the similarity with the human consensus sequences; the splicing branchpoint to 3′ splice site distance; GC content; predicted secondary structure and lengths of intron and 3′UTR of the gene. Overall, 5′ splice sites display higher similarity to the human consensus sequence over the 3′ splice site (Figure 5B). However, we did not observe a strong correlation between the fraction of spliced reads and any of the tested features, and thus none of the features analyzed thus far proved to be a reliable predictor(s) of splicing efficiency (Figure 5C).

## Discussion

In this study, we present the first analysis of pre-mRNA processing on a global scale in the thermotolerant alga, *C. merolae,* using long-read sequencing of individual RNA molecules. Our datasets yield information about *C. merolae* pre-mRNA splicing and 3′ end cleavage—two steps that have the potential to generate diverse mRNA isoforms. Using LRS, we were able to quantify the frequency of APA in *C. merolae* and the splicing efficiency of its intron-containing genes. Our results indicate frequent APA, because 63% of protein-coding genes have more than one PAS (Figure 2B). The UAAA tetranucleotide was identified as the major motif defining the polyA signal. Interestingly, nitrogen starvation slightly improved otherwise overall poor splicing efficiencies of the 27 originally identified *C. merolae* introns (Figure 4B).

Our comprehensive transcriptome-wide analysis of APA in this red alga indicate similarities with the green alga, *Chlamydomonas reinhardtii* that was found to have 40% or 68% of genes with APA in two different studies (Bell et al., 2016; Zhao et al., 2014). UGUAA was recognized to serve as the major polyA signal motif in *C. reinhardtii* and was used with a frequency of about 95% (Bell et al., 2016; Li and Du, 2014; Shen et al., 2008; Wodniok et al., 2007; Zhao et al., 2014). Our findings identified the UAAA motif as the primary polyA signal in *C. merolae* (Figure 3A), resembling the major canonical polyA signal AAUAAA used in a wide range of species, including human, mice, *Caenorhabditis elegans*, *Drosophila melanogaster* as well as budding and fission yeasts (Graber et al., 1999; Tian et al., 2005; Kamasawa & Horiuchi, 2008; Mangone et al., 2010; Ozsolak et al., 2010). In the first study attempting to predict *C. merolae* PASs based on genomic sequence and expressed sequence tags, the authors concluded that UAAA motif is present in 86.55% of transcripts and GUAA in 43.23% of transcripts (Zhao et al., 2019). This is partially consistent with our findings; we did not identify GUAA as a polyA signal motif.

The frequency of intron retention in 27 *C. merolae* mRNAs (30–90% on a per intron basis, see Figure 4A) suggests relatively inefficient pre-mRNA splicing compared with other species where introns are removed co-transcriptionally (Carrocci & Neugebauer, 2019). The significant correlation of fraction of spliced reads between our polyA+ and polyA+/− libraries (Figure 1C) suggests that *C. merolae* splicing is post-transcriptional. Thus, the long-read data agree with the first study of splicing in this organism, which revealed a large unspliced fraction by RT-PCR (Stark et al., 2015). One of the most plausible explanations for the abundant intron retention in *C. merolae* is the missing U1 snRNP and overall reduced spliceosome (Stark et al., 2015). Our 5′splice site motif analysis identified the sequence GUAAGU (Figure 5B), which is almost identical with the vertebrate 5′splice site consensus sequence that is recognized by U1 snRNP (Rosbash and Seraphin, 1991). It remains to be elucidated which factor recognizes the 5′splice site motif in *C. merolae*. Although we cannot exclude the possibility that a U1 snRNA gene has eluded sequencing or annotation, a distinct possibility is that the sequence conservation is due to base-pairing with U5 and U6 snRNAs, which exhibit unusual complementarity to 5′splice sites in *C. merolae* (Stark et al., 2015).

Intriguingly, other intron-sparse organisms, such as *Encephalitozoon cuniculi*, *Guillardia theta* and *Porphyridium purpureum*, also display low splicing efficiency (Grisdale et al., 2013; Wong et al., 2018; Wong et al., 2021). Transcriptome-wide sequencing analysis of the microsporidium *E. cuniculi,* with 37 annotated introns in a 2.9 Mb genome, revealed a low splicing rate as more than half of its introns remained unspliced in >80% of reads (Katinka et al., 2001; Grisdale et al., 2013). Comparably to in *C. merolae,* the spliceosome of *E. cuniculi* is reduced and no U1 snRNA has been identified (Katinka et al., 2001; Davila Lopez et al., 2008). Another example of an extensive intron retention is found in the 551 kb nucleomorph genome of the cryptomonad alga *Guillardia theta* that contains 17 introns (Wong et al., 2018). Finally, the 22.1 Mb genome of the red alga *P. purpureum* was predicted to contain only around 200 introns (Bhattacharya et al., 2013; Wong et al., 2021). Approximately half of its transcripts were found to remain unspliced, and the predicted U1 snRNA in *P. purpureum* is unusually long with an atypical folding pattern (Wong et al., 2021). Similar to *C. merolae,* some of the spliceosome components are reduced in *P. purpureum* (Wong et al., 2021). Therefore, reduced spliceosomes may to contribute to the poor splicing efficiencies in these organisms*.* An alternative possibility is that the high level of intron-containing transcripts is due to a decreased degradation rate, even though many RNA degradation factors were bioinformatically predicted to be encoded by the *C. merolae* genome (Reimer et al., 2017).

Based on our observations, nitrogen starvation improves splicing in *C. merolae* (Figures 4A,B) suggesting that genes with improved splicing may code for proteins contributing to cell survival in these stress conditions. Studies in another intron-sparse organism, the budding yeast *S. cerevisiae*, revealed that the accumulation of introns of specific genes helps the organism to cope with nutrient deprivation (Morgan et al., 2019; Parenteau et al., 2019). Even though the trend is opposite to *C. merolae* it implies the possibility that introns themselves might have a role in growth under normal or stress conditions. For example, the Johnson lab showed in budding yeast that some retained introns can provide alternative start codons to produce alternative protein products in response to changing environment (Hossain et al., 2016). Interestingly, *C. merolae* genes have intron-encoded AUG’s, some with the potential to serve as alternative start codons and produce shortened protein isoforms.

Altogether, *C. merolae* provides a valuable platform for understanding the molecular basis of these complex and essential processes in a simplified system. It would be interesting to use *C. merolae* as a model system to address basic mechanisms of coupling between pre-mRNA processing events, such as splicing and cleavage. A prime example is the failure of cleavage at PAS that lead to transcriptional read-through and the production of 5–45 kb long Downstream of Gene (DoG) transcripts (Hangauer et al., 2013; Vilborg et al., 2015; Vilborg et al., 2017). DoGs are transcripts produced by RNA polymerase II under stress conditions and may function as first responders that help the cell to survive. Further investigation of splicing in *C. merolae* might help to elucidate unknown details of the mechanism of splicing since its simplified spliceosome reveals the necessity or dispensability of individual spliceosome factors. Similarities in the polyA cleavage machinery are expected, based on sequence similarities in polyA signals revealed here. However, significant differences could also be expected in *C. merolae* when this machinery is more deeply investigated in future studies.

## Data Availability

The sequencing data generated in this study are deposited in NCBI SRA under the BioProject accession number PRJNA785740 (https://dataview.ncbi.nlm.nih.gov/object/PRJNA785740?reviewer=n4m3m499ajm2shl4hsm9l7e 6fc.).
